# Independent researchers in east Asian surgical research: structural barriers and potential policy pathways

**DOI:** 10.3389/frma.2026.1939323

**Published:** 2026-09-23

**Authors:** Cheng Che, Xu Gong

**Affiliations:** 1 Department of Medical Affairs and Department of General Surgery, The Third People's Hospital of Suining, Suining, China; 2 Independent Researcher, Chengdu, China

**Keywords:** East Asia, independent researchers, research evaluation, research governance, surgical research

## Abstract

East Asia is now a major center of surgical innovation. Its leading institutions have contributed to clinical care, technology adoption, and international trials. Even so, surgical research capacity, infrastructure, and academic influence remain mostly centered on major hospitals and established academic networks. Such concentration can narrow the questions asked, the methods used, and the range of people able to ask them. In this Perspective, we argue that East Asian surgical research would benefit from legitimate pathways for qualified independent researchers: professionally trained investigators who maintain substantial intellectual autonomy without holding conventional senior institutional appointments. We distinguish such researchers from amateurs by institutional affiliation, employment status, funding access, and intellectual autonomy. These dimensions, we show, operate differently in China, Japan, and South Korea, with consequences for funding eligibility, ethics review, and data access. We then outline the risks independent inquiry can carry and the safeguards that should accompany it. We propose practical measures that institutions and funders could adopt: proportionate ethics routes, transparent data-access procedures, small-grant schemes, and fairer evaluation. Our aim is to widen the pool of investigators able to meet these standards, not to lower them.

## East Asian surgical success and a structural blind spot

East Asia has made remarkable strides in surgical innovation and clinical excellence over recent decades. China's rapid adoption of robotic surgery is being investigated in bibliometric analyses, including studies of breast cancer (Ma et al., 2023); Japan has advanced minimally invasive approaches in esophageal cancer and other gastrointestinal surgery (Ozawa, 2020); South Korea has reported advances in precision treatment for early gastric cancer after non-curative endoscopic submucosal dissection (Chung, 2024). Bibliometric evidence from China suggests that such advances reflect decades of growing investment in medical education, hospital infrastructure, and translational research (Chen et al., 2024). Large institutions remain indispensable for assembling multidisciplinary teams, maintaining biobanks and registries, recruiting patients for prospective studies, delivering complex interventions, and translating discoveries into practice at scale.

However, scientific strength should not be measured only by size, output, or speed of technological adoption. Critiques of institutional concentration and metric-driven evaluation have long been central themes in science and technology studies (Hicks et al., 2015). A mature research ecosystem must also retain the capacity to question its own assumptions—a capacity constrained when career progression, access to cases and data, authorship opportunities, grant success, and professional recognition are concentrated within a limited number of institutions and senior networks (Carpenter et al., 2014).

In Japan, academic careers remain strongly anchored in university departments and institutional networks (Lawson and Shibayama, 2015); in China, a national policy document issued in 2020 found it necessary to curb the overuse of SCI-related indicators in academic evaluation (Table 1). South Korea's surgical research similarly reflects these patterns, with publication metrics dominating promotion decisions. This concentration can reduce the diversity of research questions and methods, limiting the range of perspectives brought to bear on complex surgical problems.

**Table 1:** Three institutional gates for independent surgical research in China, Japan, and South Korea: competitive funding, ethics review, and access to national health data.

Country	Funding	Ethics review	Data access	Source
China	NSFC applications must be submitted through a registered host institution, which assumes responsibility for project management and fund use.	Human-participant research requires review by an institutional or delegated regional ethics committee under the national measures issued in 2023.	No unified open national claims database; access is negotiated case by case with data holders, and human genetic resources are subject to separate regulation.	(State Council of the People's Republic of China, 2024; Ministry of Education Ministry of Science Technology of the People's Republic of China, 2020; National Health Commission of the People's Republic of China, 2023)
Japan	KAKENHI applicants must belong to an eligible host institution that administers the grant and verifies the applicant's research activity.	Specified clinical research requires review by a certified Clinical Research Review Committee under the Clinical Research Act (Act No. 16 of 2017).	NDB raw data available to approved researchers; NDB Open Data aggregate statistics freely available since 2016.	(Carpenter et al., 2014; Ministry of Health, 2017; Ministry of Health Labour Welfare of Japan, 2025; Japan Society for the Promotion of Science (JSPS), 2026)
South Korea	NRF-managed programmes are applied for through a Korean host institution; individuals cannot apply alone.	Use of NHIS data requires IRB approval or exemption before application.	NHIS customized data via the NHISS remote-access environment, with data-provision committee review and use fees.	(National Research Foundation of Korea (NRF), 2026; National Health Insurance Service of Korea (NHIS), 2026)

NSFC, national natural science foundation of China; KAKENHI, grants-in-aid for scientific research; NRF, national research foundation of Korea; NDB, national database of health insurance claims and specific health checkups; NHIS, national health insurance service; NHISS, NHIS sharing service; IRB, institutional review board.

Bibliometric analyses of surgical research in the region demonstrate concentration in particular institutions and research domains; however, such publication patterns alone cannot establish the institutional or career incentives underlying research-question selection (Kamil et al., 2023). Studies comparing successive technical refinements, retrospective analyses of single-center cohorts, and rapid applications of fashionable molecular or artificial-intelligence methods can all be useful, but concern arises when these approaches dominate at the expense of more difficult questions: which patients truly benefit from an intervention, what outcomes matter after years rather than months, whether a costly technology improves equity or merely convenience (Meara et al., 2015), how surgical quality should be judged across different care settings, and when established anatomical or oncological assumptions should be reconsidered. Such questions require time, conceptual independence, and a willingness to challenge professional hierarchies, commercial enthusiasm, or prevailing clinical routines. East Asian surgical research therefore needs not fewer major centers, but more intellectual routes alongside them.

## Independence is a research condition, not an absence of standards

The term independent researcher can create misunderstanding. It should not mean an unregulated individual working outside professional norms, nor someone claiming authority without relevant expertise. Rather, it refers to a trained investigator who retains meaningful autonomy in framing questions, selecting methods, interpreting findings, and declaring conflicts of interest. Such a person may be a clinician outside a conventional promotion track, a former academic investigator, an epidemiologist, engineer, data scientist, health economist, medical illustrator, or patient-outcomes scholar. In our view, the concept is best specified along four dimensions: institutional affiliation, employment status, funding access, and intellectual autonomy. Institutional affiliation concerns whether the investigator holds a recognized link, an appointment, visiting status, or a formal agreement, with an organization able to assume administrative and legal responsibility for the research. Employment status concerns whether the investigator depends on conventional academic employment; both affiliated and unaffiliated arrangements can support rigorous work. Funding access concerns whether the investigator can compete for support through transparent routes rather than only via a host institution's internal allocation. Intellectual autonomy, the freedom to frame questions, choose methods, and publish findings, including unwelcome ones, is the defining dimension; the other three are administrative conditions that should enable autonomy rather than substitute for it. For the purposes of this Perspective, independent researchers primarily refer to professionally qualified investigators who lack the conventional institutional appointments typically required for direct access to research funding, ethics review, or controlled research data, while recognizing that independence exists on a continuum.

Independence is compatible with collaboration and rigorous oversight. Research involving patients, identifiable health information, biological specimens, or intervention must meet the same ethical, legal, methodological, and reporting requirements regardless of who conducts it. The Declaration of Helsinki attaches its principles to the research activity itself rather than to the institutional identity of those who undertake it, and it requires that research be conducted only by appropriately trained and qualified individuals (World Medical Association, 2013). The objective is not to lower thresholds for independent researchers; it is to prevent institutional affiliation from becoming a proxy for scientific merit, ethical competence, or trustworthiness.

This distinction matters in surgery because the field is often described as an intrinsically asset-intensive. That description is partly correct. Randomized trials, advanced imaging, animal models, high-quality follow-up, and prospective registries require clinical infrastructure. But surgical science is broader than an operating room, a device platform, or a large case series—it includes anatomy and disease topology, decision science, outcomes measurement, implementation, rehabilitation, health economics, evidence synthesis, and computational modeling. Many of these areas can be advanced by small teams when appropriate data and governance pathways are available.

## Three national systems, three sets of gates

Our comparative analysis focuses primarily on China, Japan, and South Korea, three major research systems in the region, whose funding, ethics, and data-governance rules are documented in publicly accessible regulations, rather than on East Asia as a whole. Table 1 outlines how the gates described above are organized differently across these three systems. The differences matter. In China, national science-funding regulations require applicants to apply through a registered host institution, which assumes responsibility for project management and fund use; a national policy issued in 2020, meanwhile, found it necessary to curb the overuse of SCI-related indicators in academic evaluation. In Japan, Grants-in-Aid for Scientific Research (KAKENHI) applicants must belong to an eligible host institution (shozoku kikan) that administers the grant, and “specified clinical research” falls under the Clinical Research Act, which requires review by certified committees. In South Korea, requests to use National Health Insurance Service (NHIS) data must be supported by institutional review board (IRB) approval and are assessed by a data-provision review committee (Lim and Jang, 2025). Japan's National Database of Health Insurance Claims (NDB) is available to approved researchers, with aggregate NDB Open Data statistics freely available since 2016 (Sato and Yasunaga, 2023). These arrangements show that the region is not monolithic: the obstacles facing an independent investigator, and the levers for change, are country-specific.

## What independent researchers can contribute

First, independent investigators can conduct clinical archaeology: careful re-examination of evidence collected but insufficiently interpreted. Public trial datasets, open genomic resources, de-identified registries, imaging repositories, historical case series, and systematic review databases contain signals that may not fit the dominant narrative (Ohmann et al., 2017). Negative trials, discordant imaging-pathology findings, unexpected differences in long-term function, and variation in outcomes between hospitals can all generate clinically important hypotheses; classic secondary analyses of routinely collected data, which brought inter-hospital variation to the center of surgical quality assessment, are a case in point (Birkmeyer et al., 2002).

Second, they can strengthen theoretical and conceptual surgery. Surgical progress is frequently presented as a sequence of instruments and techniques. Yet important advances often begin with a revised view of anatomy, lymphatic spread, organ preservation, functional compensation, tissue repair, or the biological meaning of a surgical margin. These advances require disciplined reading, visual and spatial reasoning, model building, and explicit hypothesis generation: work that may not immediately yield large sample sizes but can define testable clinical studies suitable for staged evaluation (Hirst et al., 2019).

Third, independent researchers may provide a complementary perspective in technology assessment, particularly when they are sufficiently removed from procurement or commercial incentives. Surgical robots, energy devices, navigation platforms, implantable materials, and artificial-intelligence systems are introduced in settings where clinical aspiration, hospital competition, procurement incentives, industry partnerships, and media attention may overlap. Innovation should be welcomed, but enthusiasm is not evidence. Investigators with distance from procurement pathways can examine patient-reported outcomes, long-term complications, learning curves, opportunity costs, affordability, and access, not to oppose innovation, but to ask whether it delivers meaningful benefit to patients and health systems.

Fourth, they can facilitate interdisciplinary translation. The surgical questions most likely to be neglected are often located between departments: post-discharge recovery, financial toxicity, frailty, survivorship, regional disparities, environmental sustainability, and the transferability of results from elite centers to ordinary hospitals (Jiang et al., 2026). Independent researchers can move more easily across these boundaries and connect clinicians with statisticians, engineers, ethicists, economists, and patient representatives, a broader lens especially important in East Asia's diverse health-care settings (Kruk et al., 2018).

## Lessons from other fields

The concept of independent research is not new to surgery. In public health, independent research organizations have long played a critical role in policy-relevant evidence synthesis and surveillance. In biomedical informatics, small independent teams have made significant contributions to data standards and computational methods. In health services research, landmark multi-institutional studies on health system performance and patient safety, such as the multinational surgical safety checklist trial (Haynes, 2009), have reshaped practice across economic settings; such work has depended on cross-institutional collaboration rather than on any single employment model. The Cochrane Collaboration exemplifies how independent researchers can systematically review evidence across institutions and commercial interests (Allen and Richmond, 2011). The Patient-Centered Outcomes Research Institute (PCORI) in the United States demonstrates how targeted funding can support independent patient-centered research (Selby and Beal, 2012).

For surgery, the challenge is adapting these models to a technically demanding and culturally hierarchical field. The heavy-asset nature does not preclude independent inquiry; it simply requires different institutional arrangements. Just as open-access journals have demonstrated that peer review need not depend on institutional affiliation, surgical research can value methodological rigor over institutional reputation.

## The barriers are institutional as much as financial

The central obstacle is that independent researchers often lack a recognized entry point. Grant systems may require a formal host institution before a proposal is judged on its scientific value. Access to data may depend on informal professional networks rather than transparent criteria. Ethics review structures are usually designed around hospital employment, even for low-risk secondary analyses of de-identified or public data (Bazaz et al., 2026).

These barriers create a circular problem: investigators need affiliation to access data, data to generate preliminary work, and preliminary work to obtain funding. The result is not merely unfairness to individuals; it reduces the number of potentially valuable questions the system can examine.

The answer is not unrestricted access. Surgical data are sensitive, and governance must protect privacy, consent, security, and public trust. But access should be linked to the risk profile and quality of a proposal rather than the rank of an applicant's institution.

## Building workable pathways in East Asia

First, funders and professional societies should establish small, competitively reviewed grants for individuals and small teams. Review panels should prioritize question importance, analytical quality, and potential clinical value, not only institutional prestige or prior grant volume.

Second, regional mechanisms for independent ethical and methodological review should be developed for appropriate categories of research. Low-risk work using public, anonymized, or retrospectively de-identified data should have clear routes for determination, waiver where justified, or expedited review.

Third, data custodians should publish transparent application procedures. Secure data enclaves, remote analysis environments, standard data-use agreements, and preregistered analysis plans can reduce risk while enabling access. South Korea's National Health Insurance Service (NHIS) database provides an example of how large-scale health data can be made accessible to qualified researchers through secure remote access protocols, protecting patient privacy while enabling rigorous analysis, Japan's National Database of Health Insurance Claims (NDB) combines approved access for designated researchers with freely available aggregate statistics (NDB Open Data), a two-tier arrangement that balances privacy with public use.

Fourth, journals, conferences, and funders should minimize prestige bias. Blinded or partially blinded review cannot remove every signal of identity, but it can reduce reliance on affiliations, titles, and institutional reputation (Justice et al., 1998). Reporting checklists, protocol registration, open methods, code sharing when lawful and feasible, and explicit conflict-of-interest statements provide better grounds for confidence than a famous institutional address.

Finally, major centers should treat independent researchers as partners. Hospitals can provide clinical context and regulated data access. Independent investigators contribute external critique, methods expertise, and distance from local incentives.

## Risks and safeguards

Proposals to widen participation should not be romanticized. Independent inquiry carries real risks, and an honest account must name them. First, accountability: without an employer's quality-assurance structures, an individual investigator may lack the institutional oversight of protocol review, monitoring, and sanctions for misconduct (Fanelli, 2009). Second, privacy: secondary analyses of claims data, registries, and imaging repositories can expose personal information when handled carelessly. A breach by an unaffiliated researcher is as harmful as one by an affiliated team. Third, conflicts of interest: investigators outside institutional procurement and employment systems are not immune to commercial, reputational, or ideological influences. Distance from one incentive can coexist with proximity to another. Fourth, methodological variability: without peer infrastructure, work may suffer from unconstrained analyses, selective reporting, or overstated claims. Fifth, equity: the time, capital, and credentials that independent research demands could make it a pastime of the already advantaged rather than an opening for excluded talent.

None of these risks justifies exclusion; each justifies safeguards. The same ethical and reporting standards that apply to institutional research should apply in full (World Medical Association, 2013). These include protocol registration, explicit conflict-of-interest statements, and transparent methods and code where lawful and feasible. Data access should occur in secure enclaves or remote-analysis environments under preregistered analysis plans, with data custodians retaining the right to audit compliance. Accountability can be anchored through formal agreements: visiting-researcher status, society-based mentoring, or contractual responsibility assumed by a data custodian or professional body. With such arrangements, no investigator is beyond the reach of sanction for misconduct. Finally, funders can require evidence of methodological competence rather than institutional rank. Where appropriate safeguards are in place, the risks associated with participation by qualified independent researchers may be manageable and should be weighed against the potential epistemic costs of unnecessarily excluding them.

## Limitations

This Perspective has several limitations. First, it does not estimate the number or characteristics of independent surgical researchers in East Asia; we are aware of no comprehensive workforce data. Second, the comparative policy analysis covers only China, Japan, and South Korea. Its conclusions about institutional gates should not be generalized to other East Asian jurisdictions without further study. Funding, ethics, and data-access requirements may also differ across programs within the same country and may change over time. Third, this Perspective does not empirically establish that independent-research pathways would increase methodological diversity, innovation, or research quality.

Several of the proposed benefits should therefore be treated as policy hypotheses that warrant future evaluation. Investigators who wish to test the concentration-related claims empirically could conduct bibliometric analyses of East Asian surgical output: the proportion of publications originating from the top five or ten institutions; institutional concentration indices, such as the Herfindahl-Hirschman Index and Gini coefficients; the concentration of corresponding authorship; and the concentration of nationally funded surgical research.

## Conclusion

East Asian surgery does not face a shortage of talent, clinical volume, or technical ambition. Its greater challenge is to prevent these strengths from narrowing the range of questions its investigators are able to ask. Large institutions will continue to lead trials, translational programs, and complex care. Yet they cannot be the only places from which consequential surgical questions arise.

A research ecosystem that welcomes qualified independent investigators may contribute to greater methodological diversity, increased attention to conflicts of interest, and greater capacity to pursue questions whose value is not immediately captured by publication counts. Standards should remain high: ethical conduct, methodological rigor, transparency, reproducibility, and patient relevance. What should change is the assumption that only conventional institutional affiliation confers the right to participate in knowledge production.

Creating fair pathways for independent inquiry is a strategy for protecting originality, accountability, and resilience in East Asian surgical research. The region's next important contribution may not come only from a larger platform or a newer device. It may begin with a well-trained investigator who is free to see a problem differently and is given a legitimate opportunity to test that insight.

## Data Availability

The original contributions presented in the study are included in the article/supplementary material, further inquiries can be directed to the corresponding author.
